# Hyperoxemia and hypoxemia impair cellular oxygenation: a study in healthy volunteers

**DOI:** 10.1186/s40635-024-00619-6

**Published:** 2024-04-15

**Authors:** Bashar N. Hilderink, Reinier F. Crane, Bas van den Bogaard, Janesh Pillay, Nicole P. Juffermans

**Affiliations:** 1 Department of Intensive Care, OLVG Hospital, Amsterdam, The Netherlands; 2 Department of Critical Care, University Medical Center Groningen, University of Groningen, Groningen, The Netherlands; 3 Laboratory of Translational Intensive Care, Erasmus MC, University Medical Center, Rotterdam, The Netherlands

**Keywords:** MitoPO_2_, Hypoxia, Oxygen therapy, Hypoxemia, Hyperoxia, Hyperoxemia, Mitochondria, Cellular oxygenation

## Abstract

**Introduction:**

Administration of oxygen therapy is common, yet there is a lack of knowledge on its ability to prevent cellular hypoxia as well as on its potential toxicity. Consequently, the optimal oxygenation targets in clinical practice remain unresolved. The novel PpIX technique measures the mitochondrial oxygen tension in the skin (mitoPO_2_) which allows for non-invasive investigation on the effect of hypoxemia and hyperoxemia on cellular oxygen availability.

**Results:**

During hypoxemia, SpO_2_ was 80 (77–83)% and PaO_2_ 45(38–50) mmHg for 15 min. MitoPO_2_ decreased from 42(35–51) at baseline to 6(4.3–9)mmHg (*p* < 0.001), despite 16(12–16)% increase in cardiac output which maintained global oxygen delivery (DO_2_). During hyperoxic breathing, an FiO_2_ of 40% decreased mitoPO2 to 20 (9–27) mmHg. Cardiac output was unaltered during hyperoxia, but perfused De Backer density was reduced by one-third (*p* < 0.01). A PaO_2_ < 100 mmHg and > 200 mmHg were both associated with a reduction in mitoPO_2_.

**Conclusions:**

Hypoxemia decreases mitoPO_2_ profoundly, despite complete compensation of global oxygen delivery. In addition, hyperoxemia also decreases mitoPO_2_, accompanied by a reduction in microcirculatory perfusion. These results suggest that mitoPO_2_ can be used to titrate oxygen support.

## Background

Hypoxemia increases the risk of death in ICU patients by 50% [1, 2]. However, in 10% of all ICU patients, therapy with supplemental oxygen leads to supraphysiological arterial oxygen tensions [3]. As hyperoxemia is also associated with increased mortality [1–4], careful titration of oxygen therapy is imperative. Clinical trials have attempted to establish optimal oxygen targets but results are contradictory, partly due to the absence of a direct biomarker of oxygen toxicity and oxygen debt. Most likely, determining oxygen dose is complicated by the lack of knowledge of the effects on a cellular level [5–8].

The rationale for supplemental oxygen therapy is to prevent cellular hypoxia [6]. The effects on a cellular level are unclear due to the variable effects of oxygen on organ perfusion. Hypoxemia increases cardiac output, recruits previously closed capillaries and decreases mitochondrial oxygen consumption, which improve global oxygen delivery and lower oxygen demand [9–12]. On the other end, hyperoxemia reduces cardiac output and increases ROS production, leading to impairments in microcirculatory perfusion [13, 14]. This could potentially offset the increase in arterial oxygen content and limit oxygen extraction by tissues [1, 2]. Potentially supporting this, multiple analyses have shown an association between hyperoxemia and mortality in critically ill patients [1–3]. However, this relationship is not always present in severity-adjusted models [4]. As such, the causality of hyperoxemia-associated harm remains doubtful, also partly due to an incomplete understanding of the cellular and physiological effects of supplemental oxygen [6, 15].

The development of the protoporphyrin IX delayed lifetime technique enables the measurement of mitochondrial oxygen tension non-invasively in the skin (mitoPO2). Mitochondria are the utilizers of oxygen and therefore mitoPO2 reflects the balance between oxygen supply and demand at the most downstream level [16, 17]. Cutaneous mitoPO_2_ correlates well with organ mitoPO_2_ and responds accurately to changes in FiO_2_ or in tissue perfusion [16, 18–21]. Moreover, it also allows measurement of mitochondrial respiration (mitoVO_2_) non-invasively [22, 23].

The aim of this study was to investigate the effect of hypoxemia and hyperoxemia on mitoPO2 as marker of cellular oxygen availability in healthy human volunteers.

## Methods

### Study design and participants

This physiological cross-over intervention study in healthy human volunteers was conducted in the ICU of a teaching hospital (OLVG hospital, Amsterdam, The Netherlands). The study was approved by the institutional review board (MEC-u). Healthy human volunteers > 18 years and with BMI < 25 kg/m^2^, were screened for eligibility. Participants were excluded if they had an allergy for plaster adhesives, mitochondrial disease, skin lesions, anemia or had a history of smoking or altitude exposure (> 1000 m) in the 3 months previous to inclusion.

### Study procedures

Participants had an ALAcare patch placed on the sternum 4 h before start of the experiment and were monitored using ECG and pulse oximetry. An arterial catheter was placed for blood pressure monitoring and blood sampling. In the cross-over design, predetermined hypoxic and hyperoxic gas mixtures were delivered in a fixed order in all participants using high flow nasal cannula. After 30 min of accustoming to the setup, hypoxemia was titrated to an SpO_2_ of 75–85% for 15 min. The target SpO_2_ was achieved by titrating the FiO2 of the high-flow nasal cannula between an FiO_2_ of 9% and 12%. The flow-rate remained equal for all participants at 40L/h to ensure no rebreathing. When participants remained between an SpO_2_ of 75% and 85% during a complete minute, the FiO_2_ was set for the remainder of the hypoxic phase. After completion of 15 min, participants had a wash-out period of 45 min of breathing atmospheric air before commencement of hyperoxic gas breathing. Hyperoxic gas mixture was delivered for periods of 15 min with incremental FiO2 of 40%, 60%, 80% and 100%. After each step, clinical data was collected and measurements were done. MitoPO2 was measured using the COMET (Photonics Healthcare, Utrecht, The Netherlands). The non-invasive cardiac output was recorded continuously using pulse wave contour analysis with the volume clamp method. The sublingual microcirculation was imaged using sidestream darkfield imaging (SDF) at baseline, after hypoxemia and after hyperoxemia to prevent the mixing of hyperoxic gas mixture with atmospheric air during the hyperoxic phases [24–26].

### Measurements

#### COMET

MitoPO2 was computed from the mean mitoPO_2_ during the first 30 s of a dynamic measurement (1 Hz). MitoVO_2_ is defined as the rate of mitochondrial deoxygenation after local occlusion of circulation by applying pressure on the probe. It is calculated by automatic linear fitting of the slope using MATLAB (The Mathworks Inc). The method of performing a dynamic measurement and calculating mitoVO2 is described in detail elsewhere [27].

#### Non-invasive cardiac output

Cardiac index and systemic vascular resistance index were measured continuously throughout the experiment using the volume clamp method in the Nexfin device (BMEYE, Amsterdam, The Netherlands) as invasive arterial wave-form estimation of cardiac-output was not available in our ICU. The change between two measurements of Nexfin-CO has been shown to have very good agreement with invasive cardiac output and has been used previously to monitor hyperoxemia induced changes in cardiac output [25, 28, 29, 30].

A finger cuff was placed on the index or middle finger according to the manufacturer’s instructions. The mean of the last 2 min of each step was used for analysis. The cardiac index was used to calculate global oxygen delivery (DO_2_) was calculated using the following formula: DO_2_ = CO*(10*Hb/dl*SaO_2_ + PaO_2_*0.03). Hb, SaO_2_ and PaO_2_ were obtained from arterial blood gas analysis.

#### Sublingual microcirculation

The sublingual microcirculation was recorded using a handheld video microscope with sidestream darkfield imaging (SDF) with the MicroScan (MicroVision Medical, The Netherlands, Amsterdam). Sublingual measurements were done by one researcher trained in microcirculatory image recording. Directly after removal of the high-flow nasal cannula, SDF measurements were performed. Three anatomical sites were recorded: the medial and both lateral parts of the sublingual area. The validated AVA 4.3C software (Microvision Medical, Amsterdam, The Netherlands) was used for quality control and analysis of images [31]. AVA 4.3C automatically assesses the focus, contrast and stability of the images. Additionally, good quality captures required the presence of flow in large vessels, to exclude possible pressure artefacts. Images were only evaluated in case the quality was sufficient. The proportion of perfused vessels (PPV), proportion of perfused small vessels (PPV small), density, and the perfused DeBacker density were determined automatically by the software. The perfused DeBacker Density are all vessels with visible microcirculatory flow. The Percentage of Perfused Vessels (PPV) is calculated as the percentage of perfused vessels in relation to the total number of all vessels and for small vessels in particular (PPV small). Small vessels (capillaries) have a diameter less than 20 µm The mean values of three recordings of each parameter were used for final analysis.

### Statistical analysis

Sample size calculation is based on the expected drop in mitoPO2 during hypoxemia as predicted by a mathematical model as there are no previous data available for the expected effect size [32]. Modified Krogh equations predict that a saturation decrease from 98% to 85% results in a mitoPO2 decreases of 40 mmHg. Since we expected that homeostatic mechanisms (cardiac output increase and microcirculatory recruitment) would attenuate this mitoPO2 decrease, we set the minimum detectable difference at 20 mmHg. The standard deviation is expected to be 15 mmHg, corresponding to the sample standard deviation in healthy human volunteers [20]. The calculated sample size for a paired *t* test with a power of 90%, a significance level of 0.05, an effect size of 1.33 (minimum difference of 20 mmHg divided by the standard deviation of 15 mmHg) is 9 subjects. We also expected to see an increase of 20 mmHg in mitoPO2 in response to hyperoxia, based on previous studies in rats [33, 34].

Data is presented in mean ± SD or median (IQR) if non-normally distributed. Within-group differences over time were analyzed using repeated measures one-way ANOVA. Hypoxemia was compared with baseline. In case of significance, post-hoc tests were done with Mann–Whitney *U* test and Bonferroni correction for hyperoxia to determine at which FiO2 the changes in variables occurred. For hyperoxemia and hypoxemia, a separate linear regression model was performed for mitoPO2 and mitoVO2. Parameters which differed significantly (*p* < 0.05) during hyperoxic/hypoxic phases in the ANOVA analysis were entered into the model as independent variables. Statistical analysis was done using Rstudio (Posit, Vienna, Austria).

## Results

**Fig. 1:**
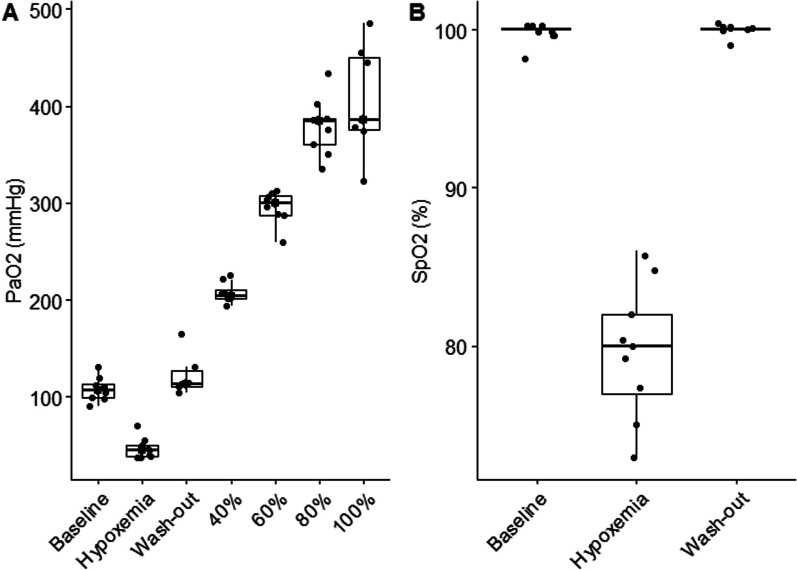
**A** PaO2 for individual experimental steps, individual datapoints and median and IQR presented. **B** boxplot of SpO_2_ for baseline, hypoxemia and wash-out steps, with individual datapoints and median and IQR

We enrolled 9 healthy volunteers, of which 6 were female. The median age was 25 (22–25) years and BMI was 21.7 (21.3–23.2)kg/m^2^. Inhalation of 10% FiO2 resulted in a decrease in PaO2 from 107 (99–113) mmHg to 45(38–50) mmHg and SaO2 from 98 (98–98)% to 80 (77–83)%, (*p* < 0.0001). Some participants noted feeling drowsy during hypoxic gas breathing, which resolved rapidly during the wash-out step. No other discomfort was noted. Hyperoxic gas breathing increased the PaO_2_ stepwise, with a plateau from 80% to 100% FiO_2_, as shown in Fig. 1A.

### Macro-hemodynamic and respiratory response

**Fig. 2:**
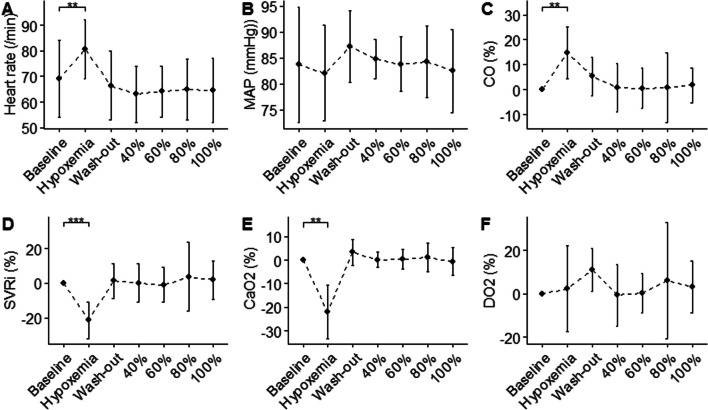
Hemodynamic variables for all experimental steps. **A** Heart-rate, **B** Mean arterial pressure, **C** % change in cardiac output compared to baseline, **D** % change in systemic vascular resistance index compared to baseline, **E** % change in arterial oxygen content compared to baseline, **F** % change in global oxygen delivery compared to baseline. Pairwise paired Wilcoxon sign-rank test compared to baseline and wash-out: *p < 0.05, **p < 0.01, ***p < 0.001,: non significance

Hypoxic gas mixture breathing resulted in an increase in heart rate from 70 (60–79) bpm to 84 (71–88) bpm (Fig. 2). This paralleled an increase in cardiac output of 16 (12–16)% from baseline and a decrease in systemic vascular resistance index of 21 (17–28)%. Whereas arterial oxygen content (CaO2) decreased with 20 (15–25)% during hypoxemia, DO_2_ was maintained when compared to baseline (*p* = 0.62). The wash-out period restored all hemodynamic indices to baseline. Hyperoxia did not induce significant changes in macro hemodynamic parameters compared to baseline. PaCO_2_ decreased significantly at 100% FiO_2_. Hypoxic gas breathing did not result in hypocapnia (Table 1). Other arterial blood gas parameters were not significantly altered during hyperoxic or hypoxic breathing.

**Table 1:** Parameters of oxygenation and microcirculation

	Baseline	Hypoxia	Washout	40%	60%	80%	100%
SpO_2_ (%)	100 (100–100)	80 (77–82)#	100 (100–100)	100 (100–100)	100 (100–100)	100 (100–100)	100 (100–100)
PaO_2_ (mmHg)	110 (99–110)	45 (38–50)	110 (110–130)	200 (200–210)*	300 (290–310)*	390 (360–390)*	390 ( 380–450)*
SaO_2_ (%)	99 (98–99)	83 (77–89)	99 (99–99)	100 (100–100)	100 (100–100)	100 (100–100)	100 (100–100)
Hb (g/dL)	12 (12–13)	13 (13–14)	13 (12–14)	13 (12–13)	13 (12–13)	13 (13–13)	13 (12–13)
Lactate (mmol/L)	0.60 (0.58–0.80)	0.80 (0.70–1.0)	0.55 (0.50–0.68)	0.60 (0.48–0.78)	0.50 (0.48–0.60)	0.50 (0.50–0.50)	0.50 (0.48–0.53)
pH	7.41 (7.38–7.59)	7.46 (7.41–7.59)	7.42 (7.39–7.47)	7.44 (7.41–7.52)	7.45 (7.42–7.57)	7.46 (7.42–7.52)	7.46 (7.42–7.47)
PaCO_2_ (mmHg)	36 (33–40)	33 (31–35)	38 (36–39)	35 (32–38)	33 (32–35)	34 (28–36)	34 (32–35)*
PPV (%)	96 (95–98)	95 (86–97)	–	–	–	–	83 (80–89)
Perfused DeBacker Density	3.6 (3.2–4.1)	3.8 (3.7–3.8)	–	–	–	–	2.4 (1.4–2.7)#
PPV small vessels (%)	100 (70–100)	100 (55–100)	–	–	–	–	75 (37–100)
Total vessel density	3.9 (3.3–4.3)	3.9 (3.7–4.2)	–	–	–	–	2.7 (1.7–3.3)
MitoPO_2_ (mmHg)	42 (35–51)	5.9 (4.3–9.0)^#^	39 (13–52)	20 (9.0–27)*	15 (13–32)*	18 (12–21)*	20 (15–25)
MitoVO_2_ (mmHg/s)	3.7 (2.9–5.0)	0.80 (0.50–1.0)^#^	3.2 (0.92–4.8)	2.0 (0.73–4.7)*	2.8 (1.7–4.4)	1.6 (0.77–3.2)*	2.4 (1.3–3.8)*

PPV, proportion of perfused vessels

*p < 0.05 compared to wash-out

^#^*p* < 0.05 compared to baseline

### MitoPO2 and mitoVO2

**Fig. 3:**
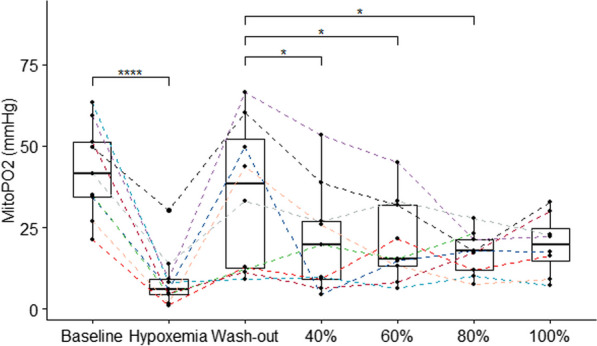
Pfaired mitoPO2 data for all participants. Kruskal–Wallis *p* < 0.001. Pairwise paired Wilcoxon sign-rank test compared to baseline and wash-out: *p < 0.05, **p < 0.01, ***p < 0.001, ns: non significance

Hypoxic mixture breathing induced a profound decline in mitoPO2 from 42 (35–51)mmHg to 6 (4.3–9)mmHg (Fig. 3). Concurrently, MitoVO2 decreased from 3.7 (2.9–5.0)mmHg/s to 0.80 (0.50–1.0)mmHg/s (*p* < 0.01). Wash-out recovered mitoPO_2_ to 39 (13–52) mmHg and mitoVO2 to 3.2 (0.92–4.8)mmHg/s.

**Fig. 4:**
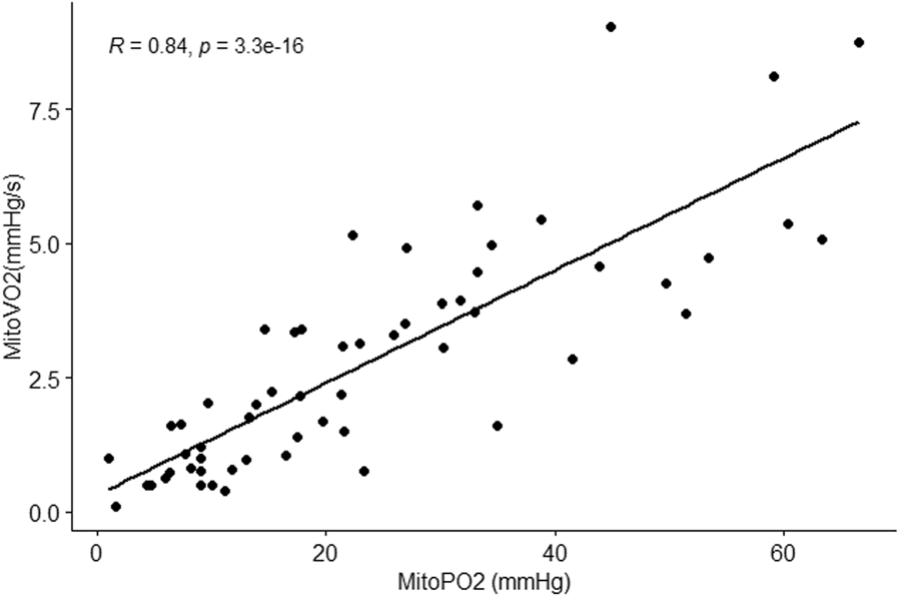
Correlation of mitoPO_2_ with mitoVO_2_ for all experimental steps. Pearson’s Rho and significance level displayed

Hyperoxic gas breathing also resulted in a median decrease in mitoPO_2_ when compared to washout (*p* = 0.03). In all participants, mitoPO2 decreased to 20 (9.0–27) mmHg when breathing 40% FiO_2_ (*p* < 0.05), which remained significantly lower compared to wash-out until 80% FiO_2_ (*p* = 0.038) (Fig. 3). Hyperoxia did not result in a significant decrease in mitoVO2 when compared to wash-out (RM ANOVA *p* = 0.2). However, mitoVO_2_ was strongly correlated with mitoPO2 ( *r *= 0.84, *p* < 0.001) during all experimental phases (Fig. 4). In linear mixed model analysis, mitoPO2 remained the only predictor of mitoVO2 with coefficient of − 0.10 (− 0.8 to − 0.12).

### Microcirculation

**Fig. 5:**
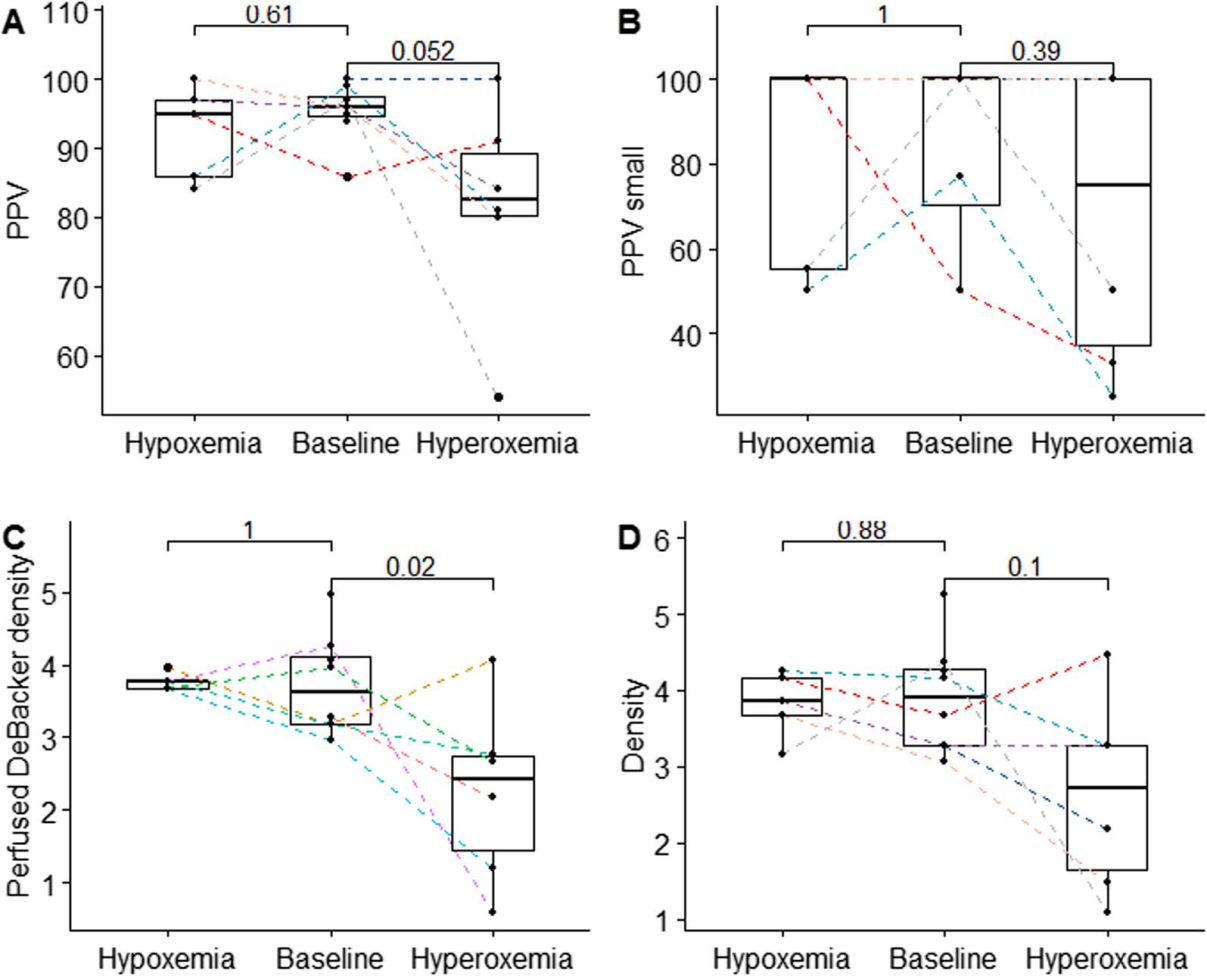
Boxplots of microcirculatory parameters at baseline, hypoxemia and hyperoxia (100% FiO_2_) with individual data points **A** Proportion of perfused vessels, **B** Proportion of small perfused vessels, **C** Perfused vessel (DeBacker) density, **D** Total vessel density. *P* values of paired-Wilcoxon sign rank test displayed

During hypoxemia, no significant recruitment of the microcirculation was observed (Fig. 5 and Table 1). During hyperoxic gas breathing, parameters of sublingual microcirculation worsened (Fig. 5). Proportion of perfused vessels (PPV) decreased from 96 (95–98)% to 80 (80–89)% at the end of the hyperoxia period. The absolute number of perfused vessels decreased in parallel, from 3.6 (3.2–4.1) to 2.4 (1.4–2.7).

### Determinants of mitoPO_2_

**Fig. 6:**
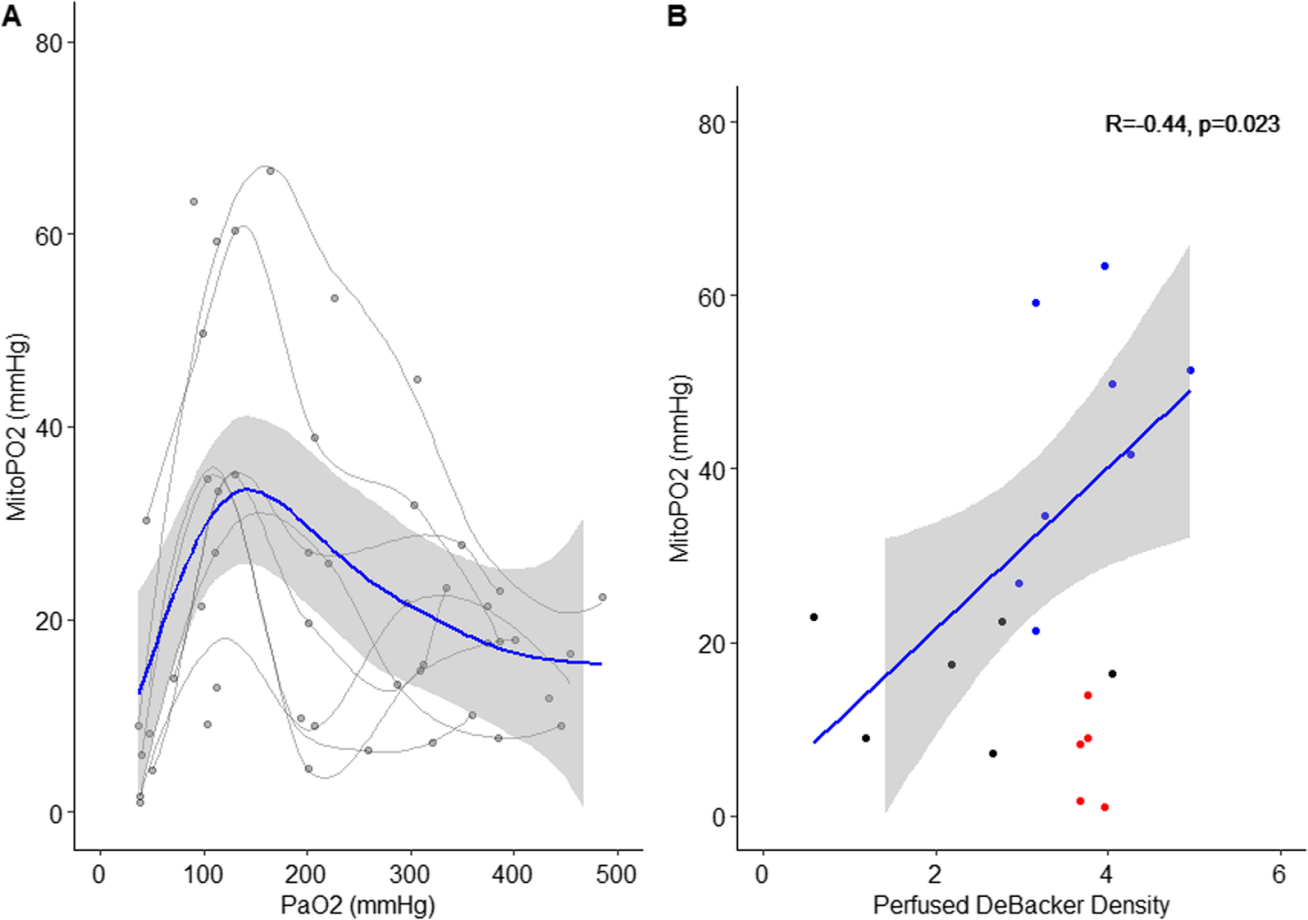
**A** Correlation of mitoPO2 and PaO2 for all experimental steps, regression line (blue) with confidence interval fitted by generalized additive model. **B** Correlation of mitoPO_2_ and perfused vessel density for all experimental steps, in red data points during hypoxemia, in blue during baseline. Linear regression fit and its Pearson’s Rho and significance level displayed for hyperoxia and baseline steps only

A linear regression model was performed to determine the influencing factors on mitoPO_2_. PaO_2_ and Perfused DeBacker density were identified as significant predictors of mitoPO_2_ during hyperoxia and baseline/wash-out (*p* < 0.05). During the hyperoxic phase, mitoPO2 was linearly correlated with PaO_2_ (*r* = − 0.44, *p* < 0.01) and the perfused DeBacker Density (*r* = 0.6, *p* = 0.023). Figure 6 shows the fitted general model with PaO_2_ and perfused DeBacker Density as explanatory variables. Inclusion of the hypoxic phase results in a non-significant correlation between perfused DeBacker density and mitoPO_2_, due to an inability of perfused DeBacker density recruitment to restore mitoPO2 to baseline values (Fig. 5C).

## Discussion

We assessed the effect of acute hypoxemia and hyperoxemia on mitoPO_2_ as a marker of oxygen debt and toxicity. Despite complete compensation of global oxygen delivery during hypoxemia, mitoPO_2_ decreased in all participants. Also, we demonstrated that hyperoxemia is detrimental to mitoPO_2_, through reduced microcirculatory perfusion.

MitoPO_2_ at baseline was 40 mmHg, corresponding to previous studies in healthy volunteers [19, 27]. MitoPO_2_ decreased sharply in acute hypoxemia. This corroborates predictions from mathematical experimental models which showed significant reductions in mitoPO2 in response to hypoxemia [32, 35]. Although intuitive, this finding is in contrast with some studies in healthy volunteers which show that indirect markers of tissue oxygenation are largely unchanged during acute hypoxemia [11, 15, 36–38]. Accordingly, it was proposed that adaptive mechanisms, such as an increase in CO and recruitment of the microcirculation could maintain oxygen delivery to cells and has led to the speculation that lower SpO2 targets could be beneficial in critically ill patients [15]. Indeed, we found an increase in CO through an increase in heart rate, causing a maintained global DO_2_ during hypoxemia. Nevertheless, the sharp decrease in mitoPO_2_ shows that hypoxemia decreases oxygen delivery into the parenchyma. The probable explanation is that in the microcirculation, the augmented blood flow above physiological levels is not beneficial during acute hypoxemia because the time for red blood cells to unload their (limited) oxygen content decreases, thus causing hypoxemic tissue hypoxia [12, 32, 39]. Although systemic DO_2_ reflects the total oxygen content carried per unit time, it does not reflect the ability of the microcirculation to unload oxygen into the parenchyma. Mathematical models and experimental studies corroborate this disconnection showing a decrease in ScvO_2_ and increase in oxygen extraction ratio during hypoxemia [32, 40]. An alternative explanation is that despite an increase in CO, the observed decrease in mitoPO_2_ is mediated by redistribution of blood flow away from the skin, kidneys, GI and liver to the heart and brain during hypoxemia [41–44]. In shock and critical illness, skin blood flow closely resembles visceral organ blood flow. However, it remains unknown whether this close relation remains during a combination of hypoxemia and shock. The detrimental effects of hypoxemia on internal organs is further supported by significant cognitive decline in healthy human volunteers during acute hypoxemia, likely representing slight cerebral oxygen debt [32, 45]. Commonly used markers of tissue oxygenation/perfusion such as NIRS and lactate, may not be suitable to ensure adequate cellular oxygenation in the context of acute hypoxemia [15].

Hyperoxemia also causes a profound decrease in mitoPO_2_, occurring at a PaO_2_ level of 200 mmHg. This occurred in parallel with a decrease in sublingual perfused vessel density. Although previous studies have shown a decrease in microcirculatory perfusion [30, 40, 46, 47], it was also shown that oxygen delivery increased with increased PaO_2_ [48–50]. This combination has led to the accepted hypothesis that an increased tissue oxygen tension induces radical oxygen species mediated damage. However, tissue pO_2_ measurements were performed using devices that disturb the integrity of the tissue. Also, the SDF technique has been unable to elucidate the downstream effect of hyperoxemia on oxygen toxicity and debt as it is not able to evaluate the oxygen content of capillaries [46, 49, 51, 52]. This study is the first to directly measure cellular oxygen availability in response to hyperoxemia and indeed demonstrates that a PaO_2_ above 200 mmHg has a detrimental effect on cellular oxygenation, at least in healthy volunteers. Of note, this cutoff value (200 mgHg) corresponds well with PaO_2_-associated mortality in critically ill patients [1, 2]. Our results show no dose-dependency of decreasing mitoPO_2_ while increasing PaO_2_, with the effect plateauing at an FiO_2_ of 40%. This corresponds to meta-analyses showing that PaO_2_ has no dose-dependent effect on mortality [1, 5]. Taken together, our findings suggest that a reduction in tissue oxygenation, through reduction in microcirculatory perfusion might account for the observed harm of hyperoxemia in hospitalized patients.

Our study has potential implications for future clinical investigations into hyperoxemia and hypoxemia. Intensivists frequently assess markers of tissue oxygenation (lactate, microcirculation) when hypoxemia is refractory. However, whereas no evidence exists that lactate reflects tissue hypoxia during hypoxemia, we demonstrate that mitoPO_2_ may be an alternative [11, 15, 37, 53]. Increasing PaO_2_ during normoxemia to attempt to increase oxygen delivery is done frequently [54]. However, this has never been empirically demonstrated to be effective and guidelines provide contradictory recommendations for supplemental oxygen therapy during normoxemia [55–60]. We show that from a PaO_2_ of 200 mmHg and above, median mitoPO_2_ is lower than the 25 mmHg threshold associated with organ failure in critically ill patients. As such, our results suggest that O_2_-supplementation should probably not exceed an upper PaO_2_ limit of 200 mmHg, as it is associated with a decrease in tissue oxygenation. However, it remains to be investigated whether a low mitoPO_2_ reflects adverse effects on cellular integrity and organ function in patients [61]. In addition, the heterogeneous effect of PaO_2_ on hemodynamics warrants further investigation of mitoPO_2_ as a biomarker of oxygen toxicity and debt for personalized titration of PaO_2_ in critically ill patients.

## Limitations

Our study has several limitations. As this was a study in healthy volunteers, results might not apply to the critically ill patient. Patients in the ICU often have disturbed Hb-O_2_ dissociation curves and impaired vascular reactivity, meaning high/low PaO_2_ and ROS could have a different effect on the visceral and skin microcirculation in patients with systemic inflammation compared to healthy volunteers. Furthermore, the coupling between skin and visceral mitoPO_2_, blood flow and microcirculation that is seen in experimental hypoxemia could potentially be absent in critically ill patients with shock.

Also, we did not control for normocapnic hypoxemia. Alkalosis was observed in most participants throughout the experiment. However, we found that decreases in mitoPO_2_ were not explained by hypocapnia as continuous variable in the mixed model but this may have been due to the limited sample size. Furthermore, gas mixture breathing was not randomized as we used a cross over study setting. It is known that both hyperoxemia and hypoxemia have long lasting effects on the microvasculature and arteriolar tone due to increased sympathetic activity, even after cessation of hypoxic and hyperoxic stimuli [62–64]. As such, we cannot exclude the possibility that exposure to hypoxia may have altered the response during hyperoxemia. However, SVR was restored during wash-out and no significant microcirculatory hypoperfusion was noted during hypoxemia. Finally, it is unknown whether a low mitoPO2 during hypoxemia and hyperoxemia reflects cellular damage and organ function. We call for clinical investigations for mitoPO_2_ as a marker of organ function during resuscitation.

## Conclusion

Acute hypoxemia decreases skin mitoPO_2_ profoundly, despite complete compensation of global oxygen delivery. Hyperoxemia decreases skin mitoPO_2_ dose-dependently through decreased microcirculatory perfusion. We identified a maximum PaO_2_ of 200 mmHg for optimal tissue oxygenation in healthy volunteers. These results suggest that mitoPO_2_ could be used as a marker of oxygen debt during oxygen therapy.

## Data Availability

The datasets used and/or analysed during the current study are available from the corresponding author on reasonable request.

## Abbreviations

CaO2: Arterial oxygen content
CO: Cardiac output
DO2: Global oxygen delivery
FiO2: Fraction of inspired oxygen
MitoPO2: Mitochondrial oxygen tension
MitoVO2: Mitochondrial oxygen consumption
PaCO2: Partial pressure of carbon dioxide in arterial blood gas
PaO2: Partial pressure of oxygen in arterial blood gas
PPV: Proportion of perfused vessels
ROS: Radical oxygen species
SaO2: Arterial haemoglobin oxygen saturation
SDF: Sidestream darkfield imaging
SpO2: Saturation of oxygen measured using plethysmography
SVRi: Systemic vascular resistance index
